# Revealing the Proximity of Concrete Specimens to Their Critical Damage Level by Exploring the Cumulative Counts of the Acoustic Emissions in the Natural Time Domain

**DOI:** 10.3390/ma17051017

**Published:** 2024-02-22

**Authors:** Dimos Triantis, Ermioni D. Pasiou, Ilias Stavrakas, Stavros K. Kourkoulis

**Affiliations:** 1 Electronic Devices and Materials Laboratory, Department of Electrical & Electronics Engineering, University of West Attica, 250 Thivon Avenue, 122 44 Athens, Greece; triantis@uniwa.gr (D.T.); ilias@uniwa.gr (I.S.); 2 Laboratory for Testing and Materials, Department of Mechanics, School of Applied Mathematical and Physical Sciences, National Technical University of Athens, Zografou Campus, 157 73 Athens, Greece; epasiou@central.ntua.gr

**Keywords:** acoustic emissions, cumulative counts, b-value, concrete, damage, criticality, natural time

## Abstract

This study aims to explore the possibility of detecting indices that could potentially provide warning about the proximity of internal damage to critical levels, beyond which catastrophic fracture is impending. In this direction, advantage was taken of the Cumulative Counts that were recorded during the mechanical loading of specimens made of either plain or fiber-reinforced concrete. The parameter adopted for the analysis was the average rate of change in the Cumulative Counts. Τhe evolution of the specific parameter was considered in the Natural Time Domain, rather than in the conventional time domain. Experimental data from already published three-point bending protocols were used. It was revealed that the specific parameter attains, systematically, a limiting value equal to unity exactly at the instant at which the load reaches its maximum value, which is not identical to the load recorded at the instant of fracture. Similar observations were made for a complementary protocol with uniaxially compressed mortar specimens. The conclusions drawn were supported by the b-values analysis of the respective acoustic data, again in terms of Natural Time. It is, thus, indicated that the evolution of the average rate of change in the Cumulative Counts in the Natural Time Domain provides an index about the proximity of the applied load to a value beyond which the specimen enters into the critical state of impending fracture.

## 1. Introduction

The monitoring technique based on Acoustic Emissions (AEs) is related to the collection, processing and proper exploitation of acoustic mechanical signals, which are produced due to the activation of internal damage mechanisms (like, for example, micro-cracking or the coalescence of microcracks) in the interior of mechanically loaded specimens, structural members or, even, whole structures. The AE signals are produced by the strain energy, which is accumulated due to the external loading and is suddenly released (assuming that the respective energy storage capacity of the material is exceeded) in the form of elastic waves. Therefore, while various types of damage mechanisms are active, acoustic signals are produced, the amplitudes of which cover a wide range of values. These signals are monitored by piezoelectric transducers (AE sensors) properly attached to the surface of the specimen (or structural member) under study. To avoid recording signals due to noise or due to external AE sources, an amplitude threshold is the first precaution. The elastic wave picked by an AE sensor is transformed into an electrical signal, amplified, processed by means of suitable software and, finally, stored in an appropriate unit.

The AEs technique has been widely applied for the investigation of the mechanical response of structures made of concrete and similar building materials [1]. It is very well documented that the temporal evolution of a wide series of parameters of the acoustic signals is closely related to the respective evolution of the mechanical properties of the specimen’s material and of the level of the internal damage accumulated (which is directly related to the remaining load carrying capacity of the specimens or the structures) [2,3]. Therefore, the AEs technique gradually became a valuable tool for scientists and engineers responsible for monitoring the deformation and integrity of structures made of concrete and other brittle structural materials, offering critical insight concerning the onset and extent of internal damage and, also, about the mechanisms responsible for the generation of the networks of micro-cracks developed in the interior of the loaded element. Indicatively, one could mention, for example, the works by Cha and Buyukozturk [4] and also the very recent ones by De Maio et al. [5], Pranno et al. [6], Machorro-Lopez et al. [7], Kontoni et al. [8] and Brahim et al. [9].

In parallel, intensive investigation is carried out in the direction of developing criteria assessing the severity of the damage of infrastructures made of concrete [10,11,12,13,14,15] using, among others, artificial intelligence tools, in an effort to quantitatively predict the remaining load carrying capacity, or, in other words, the remaining “life” of the loaded element [16,17,18,19,20,21]. In this context, quite a few interesting studies are published nowadays attempting to detect reliable signals warning about upcoming catastrophic macro-cracking. In this field, one could mention, indicatively only, the studies based on the experimental observation that the signal strength distribution of the AE signals exhibits similar characteristics to the relation between the magnitude and the amplitude of earthquakes [22,23]. Based on this observation, the b-value analysis according to the Gutenberg-Richter law [24] is nowadays widely applied in the study of AEs and the characteristics of the respective acoustic signals [25,26,27]. In-depth study of the temporal variation in the b-value in concrete specimens and structures has revealed that with increasing load a systematic decrease is observed towards values approaching the limit of one, suggesting a faster (or unstable) development of networks of micro-cracks, given that the AEs recorded are of relatively high amplitude while, simultaneously, the acoustic activity becomes more intense [26,28,29].

Among the parameters widely used in the study of the loading phase, during which micro-cracks are generated, is the rate of recording AE hits [30,31,32,33,34]. The specific parameter is recently proposed to be analyzed in terms of the F-function, which represents a sliding average value of the instantaneous values of the frequency of recording acoustic hits or of the generation of events [35,36,37,38]. Plotting the F-function in terms of the time-to-failure (along logarithmic scale) reveals that at the very last loading steps (i.e., while the applied load approaches its maximum value), the F-function starts increasing rapidly, offering a clear signal that warns about the upcoming generation of fatal macro-crack(s) and entrance of the loaded system into the stage of impending macroscopic fracture or fragmentation. 

The rate of acoustic counts (the times an AE waveform exceeds the predefined threshold) recorded during loading and that of the AE energy release are, also, used to predict the entrance of the loaded member into the stage of impending fracture, closely related to the transition from the phase of micro-cracking to that of the coalescence of micro-cracks and the onset of macro-cracking [33,34,39,40,41,42,43,44]. In general, an accelerated rate of counts is considered as an indication that the specimen or structure is approaching the fracture instant [2,34]. Along the same lines, it has been shown that the energy release rate, as it is recorded by the AEs technique, obeys a power law at the stage of intense internal damage [45]. Similar observations are reported for the cumulative AE rings, the evolution of which, at loading levels close to those causing fracture, is dictated by a strongly non-linear law [46].

In the frame of the discipline of critical phenomena [47], fracture processes are considered as critical states of a dynamic system. Therefore, seeking for indicators warning about upcoming fracture is equivalent to seeking the entrance of a loaded specimen (or a loaded structure) into a “critical state”, which is a very interesting scientific challenge. The present study is a contribution towards this exact direction, i.e., to reveal pre-failure indices predicting the entrance of a mechanically loaded dynamic system into the “critical state” of impending fracture. 

The novelty of the present study is two-fold: Firstly, advantage is taken of the Cumulative Counts (CC) recorded during loading. The specific AEs parameter (although rather disregarded) is advantageous since it eliminates uncertainties arising from the possible improper (or inadequate) setting of the AE timing parameters. Moreover, it is not related to the number of the AE hits recorded, their duration and frequency (quantities strongly related to the specific, and somehow subjective, “parameterization” of each experiment), offering, thus, a chance to draw conclusions independent of subjective decisions. The second novel aspect of this study is that the evolution of the CC is analyzed in the Natural Time Domain rather than in that of the conventional time. The term “conventional time” is used to denote the classical concept of time, as it is perceived by intuition, i.e., as it is measured by means of traditional watches or clocks, using as unit either seconds or hours or years, etc. On the contrary, the term “Natural Time” describes, exclusively, the temporal order of appearance of any element of a series of events (1st, 2nd, 3rd, etc.) and, in this context, “Natural Time” (denoted by the founders of the concept as χ [48,49,50]), is a non-dimensional quantity. The use of the concept of “Natural Time”, will be described analytically in Section 2. 

The concept of Natural Time was introduced by Varotsos et al. [48,49,50] in order to study the conditions that can lead a dynamic system into a new (critical) state, a precursor of the appearance of an extreme (catastrophic) event. The analysis of any time series in the Natural Time Domain ignores the time instant at which an element of the series is recorded, keeping only its temporal order of appearance, which is paired to the energy content of the specific element. It is well documented [48,51] that Natural Time is a useful tool that extracts hidden information from a time series of events, the energy content of which varies within broad limits. In this context, a series of recent studies takes advantage of the Natural Time concept in order to analyze the energy content of the acoustic signals (recorded while specimens or structural members are mechanically loaded) and detect the time instant at which the loaded system is entering into its critical state, thus, announcing upcoming catastrophic failure [52,53,54,55,56,57,58]. 

Along this direction, in the present study each value of the CC is associated with the respective natural time, χ, rather than with the conventional time, t. Taking into account that the CC(χ) function is monotonously increasing during any loading protocol, what is of crucial importance is the rate of its change (increase). Therefore, the parameter that is analyzed in this study is the evolution of the first derivative d[(CC(χ)]/d(χ) of the function CC(χ) from the onset of loading until the fracture of the loaded element. As it will be concluded, the above-described approach offers a detailed view (a view of increased resolution) of the processes taking place at the very last loading steps (the steps at which the applied load tends to attain its maximum value), during which the acoustic activity is strongly intensified and the rate of production of AE hits is rapidly increased. The study of the d[CC(χ)]/dχ function is achieved by means of the “sliding window” procedure, similar to that adopted recently for the analysis of the evolution of the F-function [35] in the Natural Time Domain, as it is analytically described in ref. [58]. 

## 2. Methodology of Data Representation

Assume that during the loading procedure of a specimen or structure, N acoustic hits were recorded in total. As a result, a time series of Cumulative Counts is formed including N discrete elements, as follows:

CC(t_1_), CC(t_2_), …CC(t_k_), …CC(t_N_) (1)

where t_1_, t_2_, …, t_N_ are the time instances at which each acoustic hit was recorded. Assume further that the elements of the above time series are normalized over their maximum value, which is obviously the value CC(t_N_). Then, a new time series is formed as:

CC*(t_1_), CC*(t_2_), …CC*(t_k_), …CC*(t_N_) (2)

with CC*(t_k_) = CC(t_k_)/CC(t_N_). In the Natural Time Domain, the above time series is translated to the following one:

CC*(χ_1_), CC*(χ_2_), …CC*(χ_k_), …CC*(χ_N_), (3)

where χ_k_ denotes the temporal order of appearance of the kth event, i.e., χ_k_ = k/N. 

**Figure 1:**
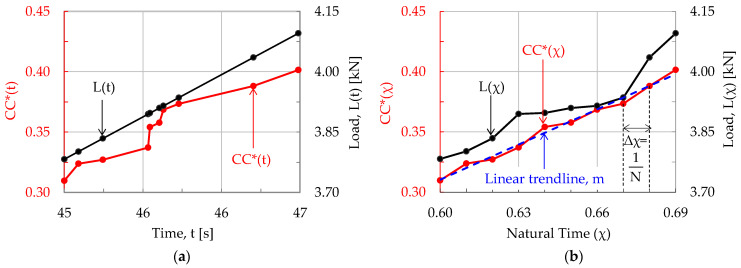
The normalized Cumulative Counts and the respective values of the applied load, in terms of the: (**a**) conventional time, t; and (**b**) Natural Time, χ.

For the above mapping procedure to become clear, let us consider a virtual experiment, implemented under, let us say, load-controlled conditions, during which N acoustic hits were recorded. Further, let us focus on a “window” with n = 10 successive acoustic hits (obviously n < N and its exact value depends on the overall hits recorded and the specific needs for resolution dictated by the analysis), which have been recorded in the time interval, let us say, 45 s ≤ t ≤ 47 s. The load applied, L, and the normalized cumulative counts CC* recorded during the specific “window” are plotted in Figure 1a. 

As is expected for a load-controlled experiment, the load–time plot is a straight line, while the (CC*–time) one is an arbitrary, monotonously increasing function. In order to draw the respective plots in the Natural Time Domain, the time interval 0 ≤ t ≤ t_f_ (t_f_ is the fracture instant) is divided into N equal subintervals with Δχ = 1/Ν. Thus, in the time window considered here, each one of the ten CC* values is mapped to the respective abscissa χ_i_, as shown in Figure 1b. In this way, an even distribution of the values of the CC*(χ) parameter is achieved along the χ-axis. Accordingly, the n values of the load at each instant t_i_, are paired to the respective CC*(χ) value and, thus, the plots shown in Figure 1b are drawn.

It is observed from Figure 1 that the evolution of the load in terms of the Natural Time parameter appears somehow distorted with respect to that in terms of the conventional time. Indeed, while in Figure 1a the (L-t plot) is a straight line, in Figure 1b it exhibits a more complex form, which is attributed to the fact that when an increased number of acoustic hits is recorded (intense acoustic activity) the load exhibits a stabilization trend (because part of the stored energy is released and, thus, the system is temporarily relieved). On the contrary, a reduced number of acoustic hits (weak acoustic activity) corresponds to a steeper increase in the load applied. The specific response will be quite often observed in the next sections of this study, in which actual experimental data will be analyzed. 

Now, using linear interpolation in Figure 1b one can calculate the slope, m, of the group of the n = 10 values of the CC*(χ) included in the specific “window”, which corresponds to the average rate of change in the CC*(χ) with respect to the parameter χ. Then, sliding the “window” by an arbitrary step Δn, one calculates the coefficient m for the overall duration of the experiment from the first time “window” to the very last one (in other words, one obtains a finite number of values of m, equal to the number of the “sliding windows” used in the procedure). The step of sliding, Δn, depends, also, on the overall number N of acoustic hits recorded. For example, one could slide the window by Δn = 1 (in case very few hits are recorded) or by a fraction of n, for example Δn = n/2 or even Δn = n (in case the number of acoustic hits recorded is very big). Obviously, the last choice (i.e., Δn = n) corresponds to a complete lack of overlap between successive “windows”.

Each m value is then paired to the average value, $\bar{\chi }$, of the Natural Times χ_i_ of the specific “window”, providing the m = m($\bar{\chi }$) function. Similarly, from the respective values of the applied load, L(χ_i_), one calculates their average value $\bar{L}$, which is also paired to the average value $\bar{\chi }$ of the Natural Times, providing the respective function $\bar{L}$ = $\bar{L}(\bar{\chi })$. 

## 3. Experimental Protocols and Results

The data used in the present study are obtained from already published experimental protocols. The specimens were prismatic (beam-shaped), notched at their central section and subjected to three-point bending. A detailed description of the specimens’ geometry, their composition and the loading scheme adopted can be found in ref. [59]. 

Advantage was taken of data from four protocols, with specimens made of (i) plain concrete (C); (ii) concrete reinforced with short poly-olefin fibers (CP/O); (iii) concrete reinforced with short poly-propylene fibers (CP/P); and (iv) concrete reinforced with short steel fibers (CM). Details about the characteristics of the fibers are found in refs. [59,60,61,62]. It is emphasized here that, since the present study focuses on temporal critical quantities and observations, the issue of data synchronization becomes a crucial one. In this context, it is mentioned that the loading frame used provides the capability to share the level of the applied load to other devices through an analogue output port. During the experiments the load was shared to the AE recording system (at a rate of 10 samples/s) as additional information when each hit was recorded (through a 2 Msample/s AE data acquisition card).

### 3.1. Protocol with Beams Made of Plain Concrete (the C-3 Specimen)

**Figure 2:**
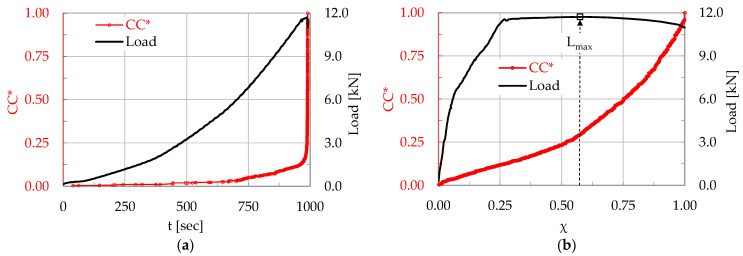
The normalized Cumulative Counts CC* and the respective values of the load (for the C-3 specimen, made of plain concrete), in terms of the (**a**) conventional time, t and (**b**) Natural Time, χ.

An overall number of N = 802 acoustic hits was recorded during a characteristic experiment (encoded C-3) of this protocol. The evolution of the CC* is plotted in Figure 2a in terms of the conventional time t, while the evolution of the CC* in terms of the Natural Time, χ, is plotted in Figure 2b. The respective evolution of the load applied is, also, plotted in both Figure 2a,b. A direct comparison of the two figures highlights clearly the superiority of the description in the Natural Time Domain (especially concerning the loading stages at levels in the immediate vicinity of the maximum load attained); indeed, the data of the AEs are spread uniformly all over the duration of the experiment, permitting thus the optimum exploitation of information that would remain hidden in the conventional time description (due to the extremely dense packing of the AEs data around the maximum load). 

The applied load attains its maximum value (equal to L_max_ = 11.7 kN) at the instant t_Lmax_ = 988 s (or, in terms of Natural Time, at χ ≈ 0.57). It is, thus, indicated that only 57% of the total number of acoustic hits were recorded until the instant of the maximization of the load. A comparable portion of the acoustic hits (equal to about 43% of the overall number of hits) was recorded during the very short time interval (of duration smaller than 2 s) between the load maximization and the abrupt load drop designating the fracture of the specimen (recorded at t_f_ = 990 s, or, equivalently, at χ = 1). 

**Figure 3:**
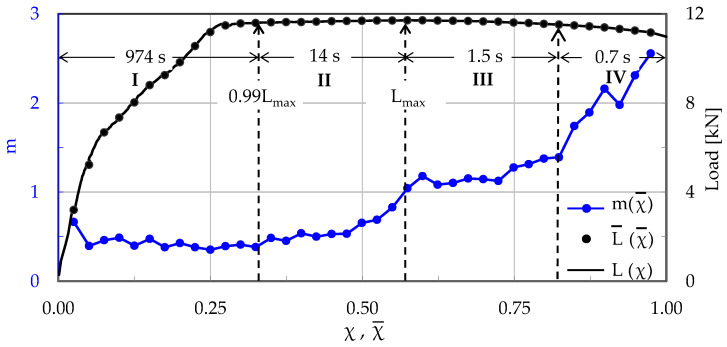
The evolution of the average rate of change, m, of the normalized Cumulative Counts, CC*, with respect to the Natural Time, in juxtaposition to the respective evolution of the applied load (for the C-3 specimen, made of plain concrete).

Taking into account that N = 802 acoustic hits were recorded in the specific experiment, it was deemed reasonable to proceed with a “sliding window” of n = 40 successive hits, using a “sliding step” equal to Δn = n/2 = 20 hits. It was proven that these choices offered quite satisfactory resolution to the analysis of the data in this experiment. In this way, a total of 39 values was obtained for the parameter m, which will now be used for quantitatively describing the evolution of the rate of the normalized Cumulative Counts in the Natural Time Domain. The above-described procedure is visualized in Figure 3, in which the parameter m is plotted versus the average Natural Time, $\bar{\chi }$, in juxtaposition to the respective evolution of the applied load. It is emphasized that all 39 linear regressions implemented for the determination of the m values were of excellent quality: the respective R^2^ coefficients were in the 0.96 < R^2^ < 0.99 range. 

Inspecting Figure 3, and based on the evolution of m, one can definitely distinguish four stages, denoted as I, II, III and IV. Stage I (0 < $\bar{\chi }$ < 0.33) accounts for the period from the onset of the loading procedure until the instant at which the load attains a value equal to about 99% of its overall maximum L_max_. During this Stage I, one-third of the overall acoustic hits produced during the specific test are recorded. The average rate m of change in the normalized Cumulative Counts is almost constant, fluctuating smoothly around the value m ≈ 0.4. Stage II (0.33 < $\bar{\chi }$ < 0.57) accounts for a time interval of a duration equal to about 14 s, during which the load level increases almost imperceptibly, finally attaining its maximum value. In this stage, the values of m increase progressively (with an increasing rate) towards a value equal to m ≈ 1. Stage III (0.57 < $\bar{\chi }$ < 0.82) is of relatively short duration (equal to about 1.5 s). The load exhibits a very weak decreasing trend. Immediately after the load is maximized, the values of m exhibit a stabilization trend and, then, they start increasing again, although rather slowly. Finally, in Stage IV (0.82 < $\bar{\chi }$ < 0.98), which is, also, of very small duration (equal to about 0.7 s), the load exhibits a decreasing trend, while the average rate m of the change in CC*(χ) increases abruptly towards its global maximum which, for the specific experiment, is equal to about m ≈ 2.6, attained at the instant of fracture. 

As a first conclusion (that will be systematically supported by the analysis of all experimental protocols), one could state the m = 1 value is a critical limit suggesting that the material of the specimen is close to exhausting its load carrying capacity and it is about to enter into its critical state, i.e., the state of impending macroscopic fracture, due to the rapid coalescence of the networks of micro-cracks that have already been generated. 

### 3.2. Protocol with Concrete Beams Reinforced with Short Poly-Olefin Fibers (CP/O-1 Specimen)

**Figure 4:**
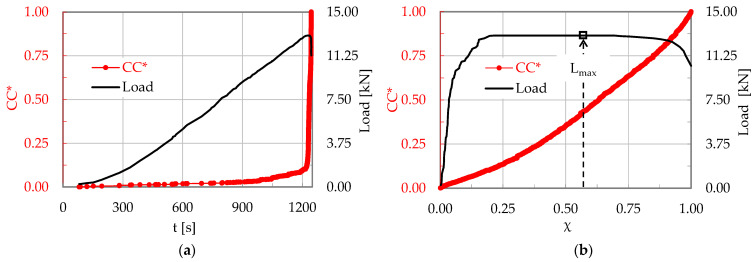
The normalized CC and the applied load (for the CP/O-1 specimen, made of concrete reinforced with poly-olefin fibers), in terms of the (**a**) conventional time, t and (**b**) Natural Time, χ.

In a typical experiment (encoded CP/O-1) of this protocol, the total number of acoustic hits that were recorded until the fracture of the specimen was equal to Ν = 889. The evolution of the normalized Cumulative Counts, CC*, is plotted in Figure 4a in terms of the conventional time t, while that in terms of the Natural Time is plotted in Figure 4b. In both figures the respective evolution of the load applied is, also, plotted. 

The fracture instant was detected at about t_f_ ≈ 1245 s. For the specific experiment, after the onset of propagation of the macro-crack front, the load was not zeroed but rather it dropped abruptly at a level equal to about 4 kN (lower than one third of the maximum value attained, which was equal to L_max_ = 13.0 kN) and, then, it was kept almost constant for a relatively long interval. In terms of the Natural Time, the applied load was maximized at the instant χ ≈ 0.52, suggesting an almost even distribution of the acoustic hits recorded in both stages of increasing and decreasing load.

**Figure 5:**
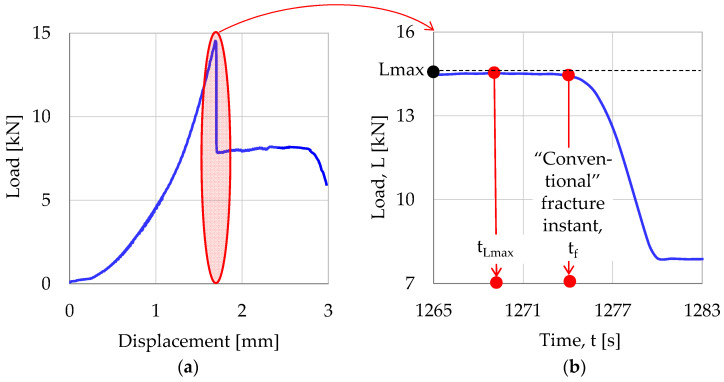
Defining the terms “Conventional” fracture and “Conventional” fracture instant.

Before proceeding to the analytical description of the experimental data for the CP/O-1 specimen, it must be mentioned here that due to the presence of the reinforcing fibers the term “fracture” is, perhaps, misleading. In fact, for all specimens made of reinforced concrete, at the instant t_f_ the load exhibits an abrupt drop but the specimens are not fragmented into two pieces, as it happened to the specimens made of plain concrete (already analyzed in Section 3.1). This response is clearly seen in Figure 5a, in which the load applied in a typical single-edge notched beam, made of fiber-reinforced concrete, is plotted versus the displacement induced during a “displacement-controlled”, three-point bending test. 

In fact, for all of these experiments, just after the abrupt load drop a macro-crack front (visible even by naked eye) starts propagating very slowly, from the crown of the pre-machined notch towards the load application line of the upper side of the beams. However, the two parts of the beam, on either side of the macro-crack front, are kept together due to the action of the reinforcing fibers. Therefore, the load applied, after its abrupt drop, is kept almost constant for an interval of relatively long duration (depending on the nature of the reinforcing fibers and, obviously, on the rate of the displacement imposed), until the macro-crack front reaches the upper side of the loaded beam. In this context, it is deemed proper from here on to use the term “conventional” fracture (Figure 5b), rather than “fracture”, and this convention is followed during the analysis of all fiber-reinforced specimens (which will be analyzed in the present section and, also, in Section 3.3 and Section 3.4).

Following the procedure of Section 3.1, and taking into account the overall number of hits recorded during this experiment, a “sliding window” including n = 40 successive hits was used, together with a “sliding step” Δn = n/2 = 20 hits. In this way, 44 values were determined for the average rate m of change in the normalized Cumulative Counts, CC*, in the Natural Time Domain. The quality of linear regressions implemented for calculating the m values was again excellent (the R^2^ coefficients ranged in the 0.97 < R^2^ < 0.99 interval). 

**Figure 6:**
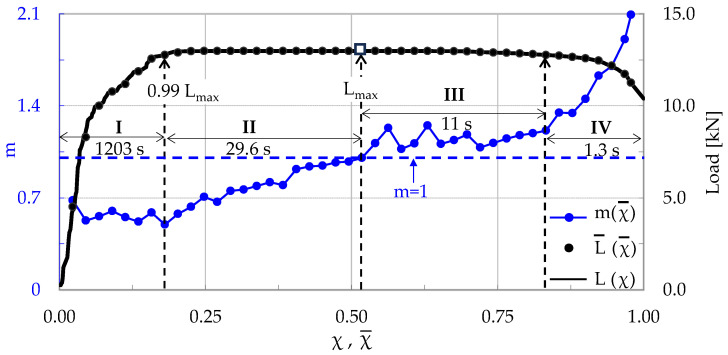
The evolution of the average rate m of the change in the normalized Cumulative Counts, CC*, with respect to the Natural Time, in juxtaposition to the respective evolution of the applied load (for the CP/O-1 specimen made of concrete reinforced with short poly-olefin fibers).

The evolution of m with respect to the Natural Time is plotted in Figure 6, in juxtaposition to that of the applied load. Again, four stages are clearly distinguishable in this test, following a pattern quite similar to that of the specimen made of plain concrete. Stage I accounts for the loading interval starting from the onset of the loading procedure until the load attains about 99% of its maximum value, or in the interval 0 < $\bar{\chi }$ < 0.18, in terms of Natural Time. It is quite interesting to note that during this stage, of a duration equal to 1203 s (i.e., about 97% of the overall duration of the specific experiment), the number of acoustic hits recorded is equal to only 18% of the total number of hits recorded during the specific test. During Stage I the m values are almost constant, fluctuating around a value equal to about m ≈ 0.6. 

Stage II, covering the 0.18 < $\bar{\chi }$ < 0.52 interval, has a duration of 29.6 s. During this stage the load increases imperceptibly by 1%, attaining its maximum value. About one-third of the total hits of the test are recorded during Stage II. The values of m start increasing steadily and almost linearly with respect to the Natural Time, reaching at the end of this stage the value m ≈ 1.0 (exactly as in the case of the plain concrete specimen). 

During Stage III (0.52 < $\bar{\chi }$ < 0.83), the load is kept almost constant (very close to its maximum level, with an imperceptible decrease of about 2%). The duration of this stage is equal to 11 s. Immediately after the load maximization, the m values exhibit a fluctuating response and, then, they start increasing again, with a relatively low rate. 

The last Stage IV (0.83 < $\bar{\chi }$ < 0.98) has a duration of only 1.3 s. The load starts decreasing towards its “conventional” fracture value (i.e., the value of abrupt load drop). As previously mentioned, the term “conventional” is used in order to emphasize that at this instant the specimen is not split into two parts but rather the (visible by naked eye) fatal macro-crack starts propagating (slowly due to the action of the reinforcing fibers, which keep the two parts of the specimen together). During this stage, the values of m increase faster, attaining a global maximum value equal to m = 2.0 at the fracture instant.

Recapitulating, it is once again highlighted that while the load approaches closely its maximum value, the average rate m of change in the CC*(χ) tends to a critical limit m = 1.

### 3.3. Protocol with Concrete Beams Reinforced with Poly-Propylene Fibers (CP/P-4 Specimen)

**Figure 7:**
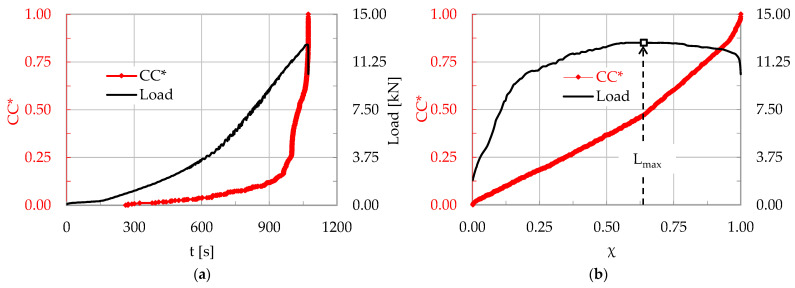
The normalized CC and the applied load (for the CP/P-4 specimen, made of concrete reinforced with short poly-propylene fibers), vs. the (**a**) conventional time, t and (**b**) Natural Time, χ.

In a typical experiment (encoded CP/P-4) of the protocol with beams reinforced with short poly-propylene fibers, a total of N = 1229 hits was recorded. The overall response is similar to that of the beam with poly-olefin fibers. The maximum load attained was L_max_ = 12.64 kN. The “conventional” fracture instant was t_f_ = 1074 s. Immediately afterwards the load dropped at 5 kN (40% of L_max_) and it remained constant while the macro-crack formed was propagating slowly, due to the presence of reinforcing fibers. The evolution of the CC* against the conventional time is plotted in Figure 7a and that against the Natural Time, χ, is plotted in Figure 7b. The evolution of the applied load is, also, plotted in both Figure 7a,b. 

**Figure 8:**
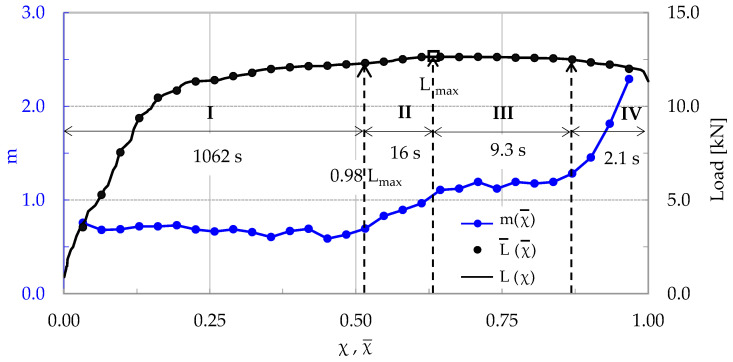
The evolution of the average rate m of the change in CC*, with respect to the Natural Time, in juxtaposition to that of the applied load (for the CP/P-4 specimen).

It is seen from Figure 7b that the load is maximized at the instant χ ≈ 0.64, suggesting that 36% of the total acoustic hits were recorded after the load maximization. For this test, a “sliding window” with n = 80 acoustic hits was used, with a “sliding step” of Δn = n/2 = 40. The difference, with respect to previous decisions, is justified by the increased number N of the acoustic hits. As a result, 39 m values were calculated, again with excellent quality in the linear regressions: the R^2^ values ranged in the 0.97 < R^2^ < 0.99 interval. Pairing these 39 values to the corresponding average value of χ, the evolution of m with respect to the Natural Time is plotted in Figure 8, in juxtaposition to that of the load.

Again, four stages can be distinguished, similarly to the specimens discussed in Section 3.1 and Section 3.2. Stage I, of a duration equal to 1062 s (i.e., about 97.5% of the overall test’s duration), accounts for the loading interval from the onset of loading until the applied load attains 98% of its maximum value, i.e., for the 0 < $\bar{\chi }$ < 0.52 interval. 

Ιt is once again highlighted that during the period that the load attains 98% of its maximum value only 52% of the total acoustic hits are recorded. The values of m during Stage I are almost constant, fluctuating smoothly around an average of m ≈ 0.65. Stage II, covering the interval 0.52 < $\bar{\chi }$ < 0.64, has a duration of about 16 s. During this stage the load increases by about 2%, attaining its maximum value L_max_ = 12.64 kN. The values of m increase steadily (and almost linearly) with respect to χ, reaching, at the end of the specific stage (i.e., at the instant of load maximization), the value m ≈ 1.05, which is very slightly higher compared to the respective values recorded for the specimens previously discussed in Section 3.1 and Section 3.2.

During Stage III (0.64 < $\bar{\chi }$ < 0.87) the load is kept almost constant with a rather negligible decreasing trend of about 1.5%. The duration of Stage III is 9.3 s. The m values initially exhibit a stabilization trend, and then they start increasing from about 1.1 to about 1.3. Finally, Stage IV (0.87 < $\bar{\chi }$ < 0.97) has a duration of only 2.1 s. The load starts decreasing, approaching the “conventional” fracture value (i.e., the value of abrupt load drop). In this stage the values of m increase rapidly, attaining a global maximum value equal to m ≈ 2.3.

Concluding, it is observed again that as the applied load tends to its maximum value, the parameter m approaches closely the m = 1 limit (in this experiment it slightly exceeded this limit), as was the case in the previous two protocols (Section 3.1 and Section 3.2).

### 3.4. Protocol with Concrete Beams Reinforced with Short Steel Fibers (CM-2 Specimen)

The last class of specimens considered in this section included notched, beam-shaped specimens, reinforced with short steel fibers. For a typical specimen (encoded as CM-2) of this class, a total number of N = 2866 acoustic hits was recorded, significantly exceeding the respective number of total hits recorded in all previously discussed experiments. This increase may be definitely attributed to the prolonged duration of the tests in this protocol, due to the increased tensile strength of the reinforcing fibers used now (it is recalled that the loading rate was kept exactly the same for all of the classes of specimens analyzed in this study). In spite of the increased number of acoustic hits recorded, the overall response of the specimens in this class is quite similar to the respective of all specimens analyzed and discussed up to now (Section 3.1, Section 3.2 and Section 3.3). 

**Figure 9:**
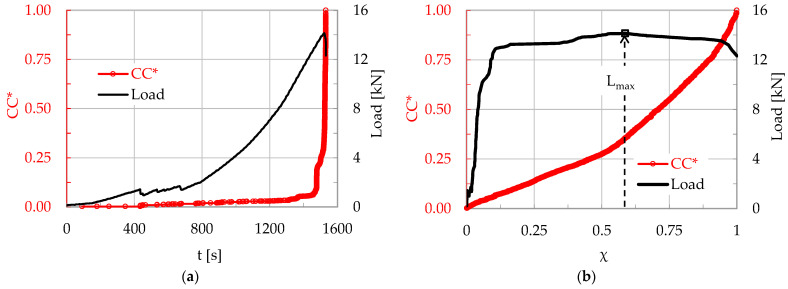
The normalized CC and the applied load (for the CM-2 specimen, made of concrete reinforced with short steel fibers), in terms of the (**a**) conventional time, t and (**b**) Natural Time, χ.

The evolution of the normalized Cumulative Counts, CC*, is plotted in Figure 9a in terms of the conventional time t. The evolution in terms of the Natural Time, χ, is plotted in Figure 9b. The respective evolution of the load is, also, plotted in both Figure 9a,b. 

The maximum load was L_max_ =14.13 kN attained at t = 1522 s. The “conventional” fracture instant was t_f_ ≈ 1532 s. The reduced load level after its abrupt drop was equal to about 70% of the respective maximum value. It is significantly higher compared to both specimens reinforced with plastic fibers, obviously due to the much higher tensile strength of the steel fibers reinforcing this specific class of specimens. 

**Figure 10:**
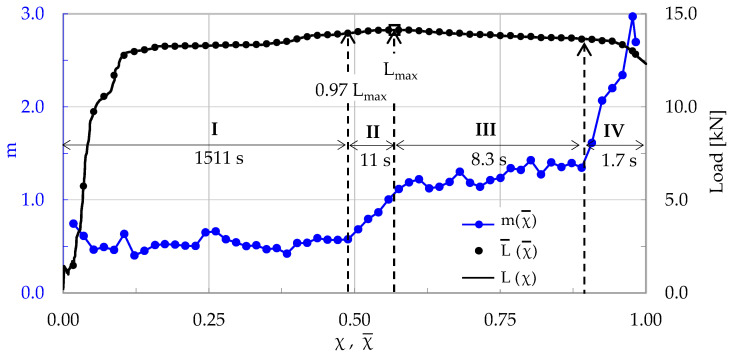
The evolution of the average rate of change in the CC*, m, with respect to the Natural Time, in juxtaposition to that of the applied load (for the CM-2 specimen).

In terms of the Natural Time, the maximum load was attained at χ = 0.59. Given that the total number of hits recorded is quite high (N = 2886), it was decided that, for the analysis of the average rate of change in the normalized cumulative counts, the sliding “window” should contain n = 100 successive hits. The “sliding step” was set equal to Δn = n/2 = 50 hits. As previously, the linear regressions implemented for the determination of the m values were excellent and the respective R^2^ values ranged in the 0.97 < R^2^ < 0.99 interval. In this way, 57 values were determined for the parameter m, which were paired to the corresponding average values of the Natural Times, χ. The evolution of m with respect to χ is plotted in Figure 10, in juxtaposition to that of the applied load. 

Exactly as in the previous protocols, four stages can be again distinguished. In Stage I, of a duration equal to 1511 s (covering 98.5% of the test’s duration until the “conventional” fracture) the m values are almost constant, fluctuating smoothly around an average value of about 0.60. At the end of this stage (covering the 0 < $\bar{\chi }$ < 0.49 interval in terms of Natural Time), the load attains almost 97% of its maximum value. During Stage II, of a duration equal to about 11 s (covering the 0.49 < $\bar{\chi }$ < 0.57 interval), the values of m exhibit a steadily increasing trend, according to an almost linear manner, attaining a value equal to about m = 1.05, exactly at the instant at which the applied load attains its global maximum. 

Stage III, of a duration equal to 8.3 s (covering the 0.57 < $\bar{\chi }$ < 0.89 interval) is characterized by a very weak decreasing trend of the applied load from its maximum value (L_max_ =14.13 kN) to a value equal to about L = 13.70 kN (i.e., a decrease of about 3% with respect to the respective maximum value). 

During Stage III, the values of m exhibit a weak increasing trend, from about 1.05 to about 1.34 with some fluctuations. Finally, Stage IV (covering the 0.89 < $\bar{\chi }$ < 0.98 interval) has a duration of only 1.7 s. The load decrease is now accelerated towards its “conventional” fracture value (that of abrupt load drop). Finally, during Stage IV, m increases quite rapidly, attaining, finally, its global maximum value equal to m ≈ 3.0.

Once again, it is definitely observed that while the load approaches its global maximum, the respective value of the average rate m of change in the CC*(χ) tends to the critical value m = 1 (for the CM-2 specimen it very slightly exceeded this limit), as was observed in all previously discussed specimens. 

## 4. Discussion

An attempt to detect possible criticality indices, or, equivalently, signs that could be considered as early warning signals about the catastrophic fracture of loaded beams was described in the present study. The topic is at the cutting edge of the respective research and is intensively studied worldwide. Indicatively only, one could mention, for example, the studies by Zhang et al. [63], and that by Chai et al. [64]. In this direction, experimental data provided by the Acoustic Emissions technique (which is the one most mature and most widely used worldwide for Structural Health Monitoring purposes) were used. The novel aspect of this attempt is that the analysis of the acoustic data was implemented in the domain of the Natural Time, rather than in that of the conventional time. The main advantage of the analysis in terms of the Natural Time is that the data are spread uniformly over the whole duration of the loading procedure, permitting deep insight into information that could remain hidden, especially in cases where a large amount of data (in the form of a time series) is densely packed within very short time intervals. 

The analysis was carried out in terms of the Cumulative Counts recorded during the whole loading procedure up to fracture. The specific parameter (although somehow disregarded in modern analysis schemes) exhibits some advantages, because it is independent from any specific setting (more or less of subjective nature) of the timing parameters of the software (and the set-up in general) of the Acoustic Emissions system, which is used in a specific experimental protocol. Indeed, the Cumulative Counts are not related to the number of the acoustic hits recorded and, also, they do not depend on the duration and frequency of the acoustic hits (quantities strongly related to the “parameterization” of the test), offering thus a chance to draw conclusions independent of subjective decisions. 

The numerical values of the most important quantities that were determined during the analysis of the experimental data (implemented in Section 3) are summarized in Table 1. Although all specimens tested had identical geometrical features, and were subjected to exactly the same loading scheme (i.e., three-point bending under constant displacement-controlled conditions), the number of acoustic hits recorded in the fiber-reinforced concrete specimens was systematically higher compared to those made of plain concrete. Especially for the specimen reinforced with metallic fibers, the number of hits recorded is almost 3.6 times higher compared to the plain concrete one. 

Another interesting characteristic that should be strongly emphasized is that a portion of only 52% to 64% of the total number of acoustic hits is generated until the instant at which the load is maximized, although the specific time interval covers more than about 99% of the overall duration of the tests. The remaining portion of the acoustic hits is recorded within the very narrow time interval, during which the load decreases, rather imperceptibly, from its maximum value to the value recorded at the instant of fracture (either it is the actual fracture or the “conventional” one). The duration of this narrow interval was only 2.2 s for the specimens made of plain concrete and it ranged between 10.0 s and 12.3 s for the specimens made of fiber-reinforced concrete. It is, thus, implied that during the very last loading steps (i.e., while the load tends to attain its maximum value) the damage mechanisms activated are quite strongly intensified and, moreover, the networks of the sources of acoustic emissions are significantly multiplied. 

**Figure 11:**
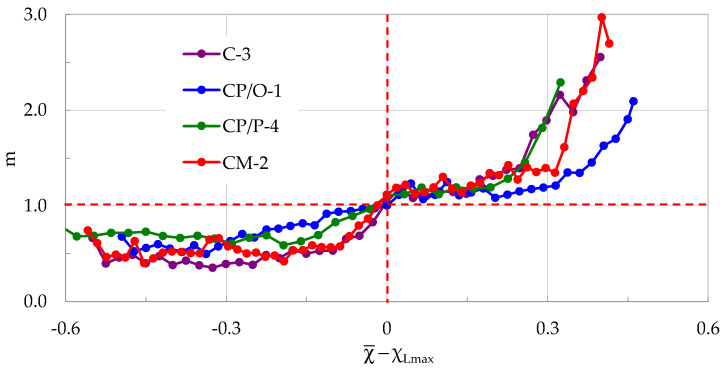
The average rate m of change in the normalized Cumulative Counts versus the ($\bar{\chi }$-χ_Lmax_) parameter of the Natural Time Domain, for all four specimens discussed in Section 3.

What is, however, the most remarkable outcome of the present study is that the evolution of the average rate of change in the normalized Cumulative Counts, m, is almost identical (from both qualitative and quantitative points of view) for all specimens tested (independently of their composition), assuming that this evolution is considered in the Natural Time Domain. The specific common feature of the specimens’ response becomes even more pronounced when the values of m($\bar{\chi }$) are plotted against the ($\bar{\chi }$-χ_Lmax_) parameter of the Natural Time Domain (it is here recalled once again that χ_Lmax_ denotes the Natural Time instant of load maximization, and does not coincide with the instant of fracture), as it is quite vividly seen in Figure 11, in which the respective plots are summarized for all four specimens analyzed in Section 3. 

It is beyond any doubt seen from Figure 11 that at the Natural Time instant $\bar{\chi }$ = χ_Lmax_ the average rate m of change in the normalized Cumulative Counts systematically approaches a critical limit equal to m ≈ 1, independently of whether the specimens are made of plain- or reinforced-concrete. For the interval $\bar{\chi }$ < χ_Lmax_ it holds, systematically, that m < 1, while for $\bar{\chi }$ > χ_Lmax_ it holds that m > 1. In other words, it could be conceived that the value m≈1 is a criterion designating the transition of a mechanically loaded system (in this case the loaded notched beam) from a stable state (m < 1) to a non-stable one (m > 1), which, in a case where the loading procedure is not interrupted, will inevitably lead to macroscopic failure. 

Furthermore, it is concluded that m values exceeding 1.5 are associated with an increased rate in the generation of AEs, which are characterized by increased numbers of counts. This combination signals the transition from micro- to macro-cracking, with a fatal macro-crack front propagating from the crown of the pre-machined notch to the interior of the specimens’ volume in the direction of the load application point. The specific statement is well validated in a very recently published paper (with specimens of identical composition to the ones studied here), the aim of which was to study the spatiotemporal evolution of the position of the sources of the AEs [59]. 

The above-mentioned criterion m ≈ 1, signaling the entrance of the loaded system into the critical state of impending fracture, appears to be independent from the total number of the hits recorded during a specific experiment. This is another fact that supports the use of counts as a quantity to be studied under the Natural Time concept relieving data from the probable subjective parameterization of the AE system. In the direction of supporting this statement, an extended time interval will be now considered for the specimens made of fiber-reinforced concrete, taking advantage of the fact that for these specimens the loading procedure is not terminated at the instant “conventionally” considered as the fracture one, i.e., the instant of the abrupt load drop. As it was already mentioned, for these specimens, after the onset of macroscopic crack propagation (and the associated load drop) the specimen is still capable of undertaking load (obviously at a lower level), which is kept almost constant for a long time interval. During this interval, the macro-crack front propagates very slowly, while the reinforcing fibers keeping the two parts of the beam together are gradually either broken or pulled-out from the concrete mass. In other words, and in spite of the fact that the macro-crack front is already formed and is propagating, there are still damage mechanisms active (both of Mode-I and Mode-II), generating acoustic hits. It is, thus, interesting to study the evolution of the average rate of change in the normalized Cumulative Counts in the period t > t_f_ (i.e., after the “conventional” fracture instant), during which the two parts of the beams are kept together exclusively by reinforcing fibers. 

**Figure 12:**
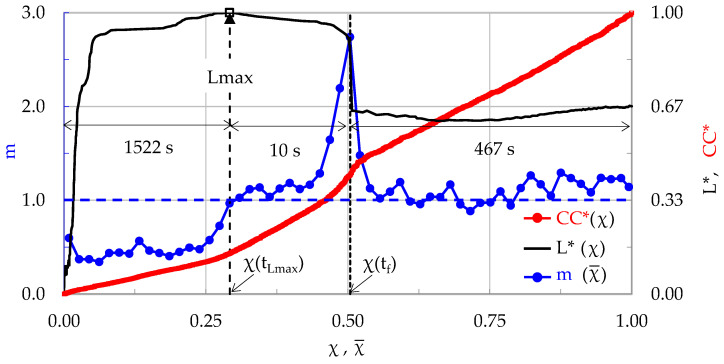
The average rate of change in the normalized Cumulative Counts, m, for an χ-interval extending beyond the “conventional” fracture instant t_f_, in juxtaposition to that of the normalized load and the normalized number of Cumulative Counts CC* (for the CM-2 specimen).

As a first example of this attempt, the CM-2 specimen (Section 3.4) is discussed. The values of m, of the normalized load L* = L/L_max_, and of the normalized Cumulative Counts CC*, are plotted in Figure 12 in terms of Natural Time, for an expanded χ-interval, extending well beyond the “conventional” fracture instant (it is noted that a direct comparison of Figure 6 and Figure 12 is not possible, since the abscissa of Figure 12 corresponds to a wider time interval). In order to plot Figure 12, the value χ = 1 was assigned to the instant t ≈ 2000 s, so that the instant t = t_f_ is assigned to χ(t_f_) ≈ 0.5. In other words, a number of 2N hits (N is the number of hits recorded until the instant t_f_) are now included in the 0 < χ < 1 window, or, equivalently, N additional hits recorded after t_f_ (i.e., while the load is almost constant equal to about 9 kN for the specific specimen), are included in the analysis. 

In order to plot Figure 12, a “sliding window” including n = 100 successive acoustic hits and a “sliding step” with Δn = n/2 = 50 hits were used. In this way 57 values were calculated for the average rate of change in the normalized Cumulative Counts, m. The critical instants χ(t_Lmax_) and χ(t_f_) are depicted in Figure 12. It is seen that the evolution of m is similar to that shown in Figure 6, and even more interesting, its value m at the instant of load maximization is, again, equal to almost 1 (in fact, it is equal to m ≈ 0.98).

Immediately after the load maximization instant, t_Lmax_, an interval of rapid increase in m follows. Its duration is equal to about 10 s and it is terminated at the instant of “conventional” fracture, at which m attains its global maximum equal to m = 2.74. After the “conventional” fracture, the values of m decrease rapidly towards the m = 1 limit, ranging with some strong fluctuations in the 0.9 < m < 1.3 interval. Concerning the evolution of the normalized Cumulative Counts, it is seen that after t_f_ it changes its curvature from positive to almost zero. It is, thus, indicated that, in spite of the fact that the macro-crack is already propagating, some damage mechanisms (of different nature) are still active and the average rate of the generation of acoustic hits is almost constant. 

**Figure 13:**
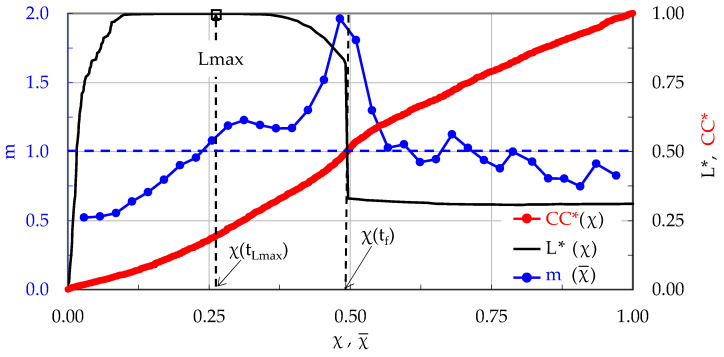
The average rate m of change in the normalized Cumulative Counts, for an χ-interval extending beyond the “conventional” fracture instant t_f_, in juxtaposition to that of the normalized load and the normalized number of Cumulative Counts CC* (for the CP/O-1 specimen).

Adopting the reasoning previously analyzed for the CM-2 specimen, the same quantities as those of Figure 12 (m, CC* and L*) are plotted in Figure 13 for the CP/O-1 specimen. It is seen from Figure 13 that at the instant of load maximization m again attains a value quite close to the critical limit of m = 1 (in fact, it is equal to m = 1.08). Immediately after the “conventional” fracture instant, the load decreases (almost instantaneously) to a value equal to about one-third of the maximum one. The average rate m of change in the CC*(χ) is now reduced relatively rapidly from its maximum value (equal to m ≈ 2.0) towards values approaching the critical limit m = 1, with some minor fluctuations and a slowly decreasing trend. As observed for the CM-2 specimen, the evolution of the normalized Cumulative Counts changes its curvature from positive to slightly negative (approaching an almost linear response). It is, thus, indicated again that damage mechanisms are still active (the fracture of fibers and pull-out of fibers from the concrete mass), while the macro-crack is propagating and the average rate of change in the Cumulative Counts is almost constant. 

**Figure 14:**
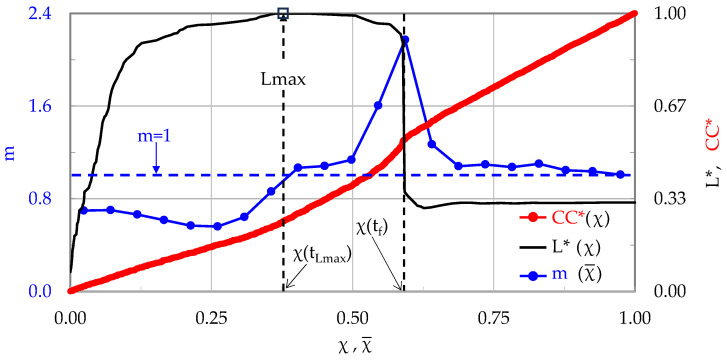
The average rate of change in the normalized Cumulative Counts, m, for an χ-interval extending beyond the “conventional” fracture instant t_f_, in juxtaposition to that of the normalized load and the normalized number of Cumulative Counts CC* (for the CP/P-4 specimen).

The response of the CP/P-4 specimen, plotted in Figure 14, is almost identical to that of the CP/O-1 specimen, with only some quantitative differences of minor importance.

As a next step, it was decided to check the conclusions drawn from the study of the evolution of the average rate of change in the normalized Cumulative Counts, m, by considering the evolution of another parameter, related to the acoustic activity (which is nowadays widely used worldwide, especially in the discipline of Earthquake Engineering), namely, the evolution of the b-value. The analysis is implemented by adopting the approach proposed by Gutenberg and Richter [24], again in the Natural Time Domain. 

**Figure 15:**
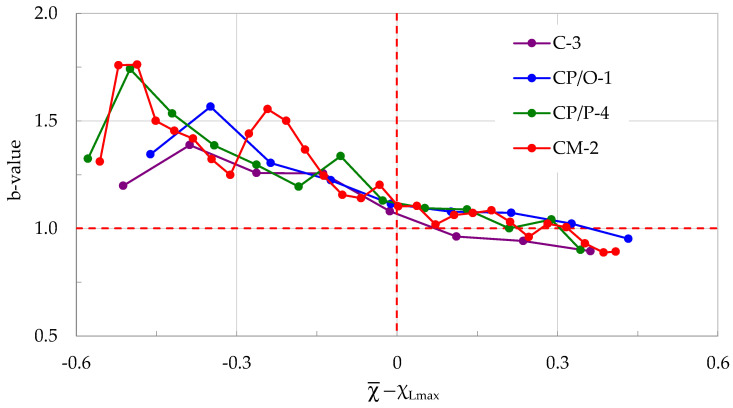
The b-values versus the ($\bar{\chi }$-χ_Lmax_) parameter of the Natural Time Domain, for all four specimens discussed in Section 3.

In this direction, the overall number of hits recorded in each one of the four experiments of Section 3 were divided into groups, each one containing approximately n = 100 successive acoustic hits. For each group the b-value was calculated. Then, using the “sliding window” approach with a “sliding step” of Δn = 100 successive hits, all of the b-values were determined up to the fracture instant (either “conventional” or actual). Each one of the b-values was paired with the respective average value $\bar{\chi }$ of the Natural Time. For the presentation of the results, the motive adopted for Figure 11 was followed, and, therefore the b-values were considered versus the ($\bar{\chi }$-χ_Lmax_) parameter. In this way, the evolution of the b-values is plotted in Figure 15 for all four experiments of Section 3.

It is clearly seen from Figure 15 that the b-values exhibit a systematic decreasing trend, almost from the onset of the loading procedure until the load attains its maximum value, i.e., in the interval $\bar{\chi }$ < χ_Lmax_. This interval corresponds directly to Stages I and II of the respective evolution of m (Section 3). For $\bar{\chi }$ ≈ χ_Lmax_, the b-values of all four specimens are equal to about b-value = 1.1, closely approaching the critical limiting value b-value = 1 [65]. 

The statement that loaded systems for which the b-values tend to the b-value = 1 limit approach the critical state of impending macroscopic fracture, is well documented in the literature [24,25,66]. As it was already pointed out in Section 3, at this instant the average rate m of change in the normalized Cumulative Counts has already attained its respective critical limit m≈1. From this instant on (i.e., for $\bar{\chi }$ > χ_Lmax_), the b-values are closely packed in a narrow band around the critical limit b-value = 1, with a very slight decreasing trend. On the contrary, during this interval the values of m increase rapidly, especially while the load starts decreasing towards the value corresponding to the “conventional” fracture. 

As a final step, the method introduced here for assessing the acoustic activity, i.e., by considering the evolution of the average rate m of change in the normalized Cumulative Counts in the Natural Time Domain, is applied to the data gathered from a different protocol (regarding both the shape of the specimens and the loading scheme). In this protocol, prismatic specimens (dimensions 50 mm × 50 mm × 70 mm), made of mortar, were subjected to uniaxial compression according to a load-controlled scheme, at a constant rate of 0.28 MPa/s. An analytical description of this protocol can be found in ref. [66], in which details are given about the preparation of the specimens and their mechanical response. 

**Figure 16:**
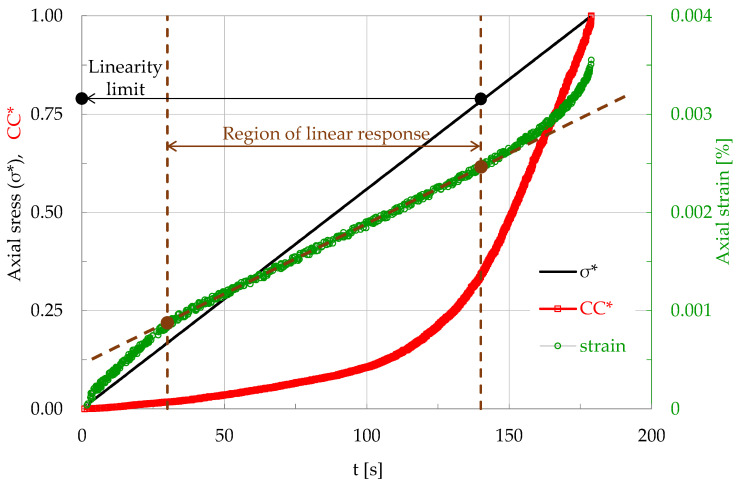
The evolution of the normalized axial stress σ*, the normalized Cumulative Counts CC* and the axial strain, in terms of the conventional time, t, for a prismatic specimen made of mortar, subjected to uniaxial compression under load-controlled conditions.

During a characteristic test of this protocol, N = 3335 acoustic hits were recorded for the overall loading procedure until the fracture of the specimen. The duration of this test was equal to 179 s and the Uniaxial Compressive Strength (UCS) was equal to σ_f_ ≈ 50 MPa. The evolution of the applied stress (normalized over the UCS) and that of the normalized Cumulative Counts, are plotted in Figure 16 in terms of the conventional time, t. In the same figure, the axial strain developed (as it was measured by means of an electrical strain gauge attached to one of the specimen’s lateral surfaces) is also plotted. As it is expected, the load curve is a straight line (recall that the experiment was implemented under load-controlled conditions). The respective plot for the strain is not linear for the total duration of the test. Its linearity region is restricted to a (relatively wide) interval ranging from about 30 s to about 140 s. The linearity limit for the specific specimen was equal to about 40 MPa. After this interval, the response of the specimen becomes strongly non-linear. 

In order to investigate the response of the specific specimen in the Natural Time Domain, 33 values were calculated for the average rate m of change in the normalized Cumulative Counts, using a “sliding window” with n = 100 successive acoustic hits and a “sliding step” of Δn = 100 successive hits. The linear regressions implemented for the calculation of the m values were also excellent for this protocol, and the respective R^2^ values ranged in the 0.97 < R^2^ < 0.99 interval. 

**Figure 17:**
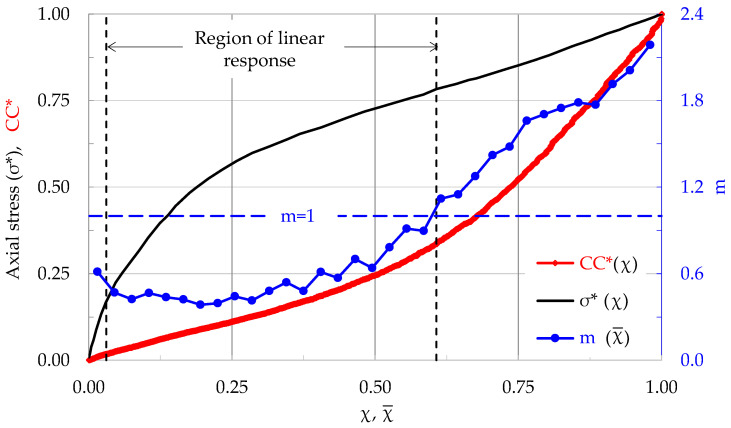
The evolution of the normalized axial stress σ*, the normalized Cumulative Counts CC* and, m, in terms of the Natural Time, χ, for the specimen discussed in Figure 16.

The evolution of m in terms of Natural Time is plotted in Figure 17, together with that of the normalized stress and the normalized CCs. It can be seen that for χ < 0.29, m is constant. Then, it increases systematically until the fracture of the specimen. The maximum value attained was m ≈ 2.2. However, what is to be most emphatically highlighted is that m attains its limiting value m = 1, almost simultaneously with the termination of the specimen’s linear response, which for the specific experiment corresponds to $\bar{\chi }$ ≈ 0.6. 

Recapitulating, it is indicated, also from this complementary protocol, that the value m = 1 signals a transition of the loaded system from a stable state (that of linear response) to a “new” state (for this case that of non-linearity). This “new” state is closely associated to non-reversible phenomena (from the thermodynamical point of view), or, in other words to phenomena related to accelerated internal damage of the specimen’s material.

## 5. Conclusions

In this study, advantage was taken of the Cumulative Counts in an attempt to explore the existence of characteristics that could be considered as pre-failure indices, adopting a procedure that is summarized as follows: the Cumulative Counts recorded during a series of previously published experimental protocols (with notched, beam-shaped specimens, made of either plain or fiber-reinforced concrete, loaded under “displacement-controlled” three-point bending), were normalized (over the respective maximum value), and then they were “translated” to the Natural Time Domain. The average rate of their change, in terms of the Natural Time, χ, was studied by introducing the parameter m. 

The analysis of the experimental data in terms of m, revealed that the loading procedure may be systematically divided into two discrete phases (a) and (b), independently of whether the specimens are made of plain or fiber-reinforced concrete. Phase (a) (including Stages I and II, described in Section 3) covers the vastly larger interval (about 99%) of the duration of any experiment, from the onset of loading up to the instant that the load is maximized. During this interval the values of m are lower than the m = 1 limit. Initially they are constant, and then they start increasing, almost linearly, while the load tends to be stabilized in the immediate vicinity of its maximum value. 

Phase (b) (including Stages III and IV, described in Section 3) accounts for the very short time interval from the maximization of the load until the fracture of the specimen (or, more, precisely, until the onset of the propagation of the front of the fatal macroscopic crack, either this propagation is instantaneous (plain concrete) or very slow (fiber reinforced specimens)). During Phase (b) the load decreases imperceptibly. Phase (b) is of extremely short duration (about 1% of the overall duration of the experiment), however, the acoustic activity is here quite intense and almost half of the overall acoustic hits recorded are generated during this interval. The values of m are now systematically higher than the m = 1 limit, with an increasing trend, which is initially very weak and then it becomes rapid as fracture approaches. During this last part of Phase (b) (the stage of rapid increase in m (coinciding, in fact, with Stage IV of Section 3), the m values range in the 2 < m < 3 interval, while the respective b-values have already reached their critical b-value = 1 [24,25,66].

In general (without any exception, at least for the specimens of the three-point bending protocols analyzed in this study), it can be safely stated that the value m = 1 is attained almost simultaneously with the maximization of the load applied. 

Concerning the complementary protocol with the uniaxial compressive loading of prismatic mortar specimens, it was concluded that the loading procedure can be again divided into two discrete phases. Phase (a) accounts for the period of the experiment during which the mechanical response of the material is linearly elastic. During this phase, the values of m are lower than the m = 1 limit. The limiting value m = 1 signals the termination of the linear response and the transition to the non-linear one, which is closely associated to severe non-reversible damage phenomena. During the phase of non-linear response, the values of m exhibit an accelerated increasing trend. 

Before concluding, it is mentioned once again that there are quite a few parameters that are extracted from acoustic emission signals. Each one of them provides interesting insight into the damage phenomena taking place within the mass of the loaded specimen. Moreover, each one of them is characterized by specific advantages and, also, limitations. In this study, it was decided to use Cumulative Counts as an alternative tool, taking into account that the specific motive of analysis of the AE data is somehow more objective, since (as it was mentioned in the Introduction) it eliminates uncertainties rising from a possible improper setting of the AE timing parameters and it is also relieved from any subjective “parameterization” of each experiment. In addition, it must be emphasized that the analysis of acoustic signals in terms of the “Natural Time” has been already attempted for other AE parameters like, for example, the energy content of the acoustic signals or the average frequency of generation of acoustic events [54] and the results are quite encouraging. The same is true for the analysis of the Pressure Stimulated Currents (PSCs) (emitted while loading specimens made of brittle materials) in terms of the “Natural Time” concept [56].

Coming to an end, it can be stated that the instant at which the value of m attains, for the first time, the critical limit of m = 1, it signals the close approach of the loaded specimen to a new, critical state, namely, the state of impending catastrophic fracture. As mentioned, m approaches the same value (m = 1) for a second time, namely, during the stage following the abrupt load drop. However, this is of minor importance for Structural Health Monitoring purposes since the specimen (or the individual structural member) has already failed: a macroscopically visible (by naked eye) crack-front has already started propagating. In this context, the m = 1 criterion may be considered as a potential pre-failure index (of practical applicability), warning about upcoming disastrous events. Obviously, the conclusions of the present study are to be further validated by additional experimental studies with specimens of more complex geometry under more sophisticated loading protocols, for example, “cyclic” loading, either in the form of a few repetitions of a given loading scheme or in the form of low cycle fatigue or even high (and ultra-high) cycle fatigue. The authors’ team is currently working on a protocol for this purpose, with specimens consisting of marble blocks joined together by means of threaded titanium bars (these specimens simulate multi-fragmented and restored epistyles of the Parthenon Temple on the Acropolis of Athens). Although the research is in progress, preliminary results already available clearly suggest that the temporal evolution of the parameter m is quite similar to the one described in this paper. 

## Figures and Tables

**Table 1:** Synopsis of the numerical values of some critical parameters.

Parameter	Plain Concrete	Reinforced Concrete		
	C	CP/O	CP/P	CM
Total number of AE hits recorded (0 < t ≤ t_f_)	802	889	1229	2886
Hits recorded in the 0 < L ≤ L_max_ interval	457	462	786	1662
Percentage of hits in the 0 < L ≤ L_max_ interval	57%	52%	64%	57%
Value of m for L ≈ L_max_	1.04	1.00	1.06	1.05
Maximum m value	2.6	2.1	2.3	2.7
Duration of the (t_f_ − t_Lmax_) interval	2.2	12.3	11.4	10.0

## Data Availability

Data are available upon request.
